# Mimetics of in-cell and subcellular crowding and solvation for protein folding

**DOI:** 10.1042/BST20250108

**Published:** 2026-04-30

**Authors:** Edward Knab, Ume Tahir, Caitlin M. Davis

**Affiliations:** Department of Chemistry, Yale University, New Haven, CT 06511, U.S.A.

**Keywords:** Cellular Mimetic, Chemical Interactions, Macromolecular Crowding, Protein Folding

## Abstract

Historically, fundamental principles of protein folding were extracted from dilute *in vitro* experiments that disregarded the complexity of the cell interior. It is now well-established that the cellular environment modulates protein behaviors. Discrepancies between protein properties measured *in vitro* and in-cell can be disentangled using mimetics that are designed to reproduce cellular interactions* in vitro*, steric crowding interactions and non-steric sticking interactions. Here, we review recent advances in the development and application of cellular mimetics of in-cell protein folding, with a focus on replicating diverse cell types and cellular compartments. Steric crowding interactions are typically mimicked using inert polymers; coupling these with giant unilamellar vesicles or phase separation allows for the creation of a cell- or organelle-like environment. Mimetics of non-steric chemical interactions must incorporate features of the chemical environment being mimicked. These range from buffers containing physiological concentrations of salt and small molecules to dilute lysates derived from the relevant cell type and/or organelle. Such mimetics of steric and non-steric interactions have greatly aided our understanding of in-cell protein folding. Mimetics can further approach biological accuracy through mixtures that simultaneously account for steric and non-steric interactions. Mimetic mixtures are important because they provide a convenient and cost-effective means to predict protein behavior in diverse cellular environments, which may benefit high-throughput applications, such as screening therapeutic candidates or training machine learning-based in-cell protein structure prediction models.

## Introduction

Much of our understanding of protein folding, assembly, and function is extracted from protein experiments performed *in vitro*. Traditionally, these studies have used dilute buffer solutions designed to stabilize the folded structure of the protein of interest and/or selected for their compatibility with the planned experiments. Less emphasis was placed on replicating interactions with the cellular environment. However, the cellular environment is dynamic, heterogeneous, and packed with other macromolecules, small metabolites, and ions that can modulate protein behaviors. Early studies performed in cells noted discrepancies between protein behaviors measured *in vitro* and *in cellulo*. For example, inside the cell, proteins rotationally and laterally diffuse slower [1–3], are thermodynamically more or less stable [4–6], and may exist in a different state altogether [7]. Thus, over the past few decades there has been a push to better understand how the cellular environment affects proteins’ structures, functions, and dynamics. The cellular environment may chemically modify a protein through post-translational modifications; these play an important role in tuning the in-cell properties of a protein, including aggregation state, subcellular localization, and proteolytic stability [8,9]. Thus, accounting for such modifications may be critical for understanding the in-cell behavior of affected proteins. In the present review, we focus on how physical interactions in the cellular environment affect protein behaviors.

**Figure 1:**
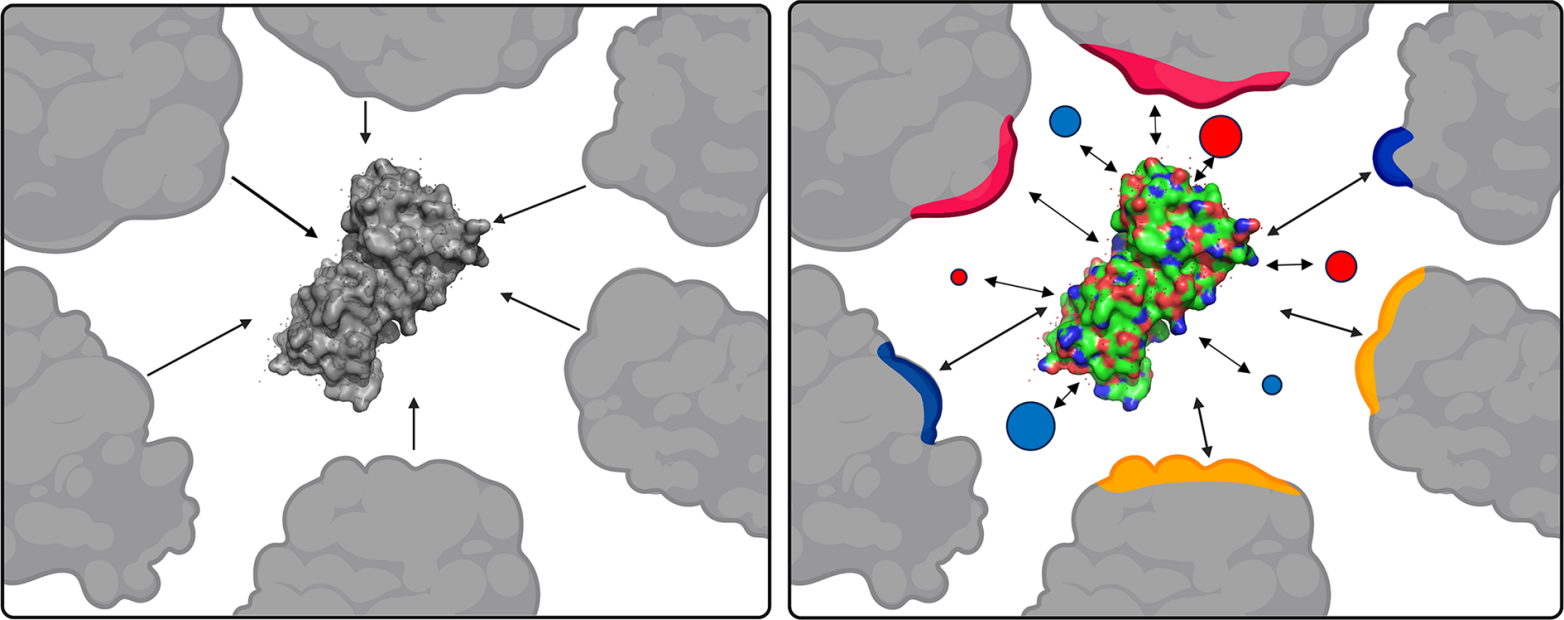
Cartoon representation of steric (left) and non-steric (right) interactions. Positive (red), negative (blue), and hydrophobic (yellow) interactions can be attractive or repulsive, and the protein can interact with macromolecules as well as charged metabolites and ions. Protein surface (PDB: 3KZ3) visualized with PyMOL [21]; other structures were created in BioRender.com and assembled in PowerPoint.

Discrepancies between protein behaviors measured *in vitro* and *in cellulo* can be broadly attributed to two fundamental physical interactions: steric and non-steric interactions (Figure 1). Steric interactions arise from the space that macromolecules occupy in the crowded cellular environment. Ogston first proposed that the presence of large macromolecules reduces the volume accessible to other solutes through excluded-volume effects [10]. He and others later validated this concept experimentally [11,12]. Building on this framework, Minton and Wilf demonstrated that excluded volume influences both the structure and function of enzymes [13]. In Minton’s model, macromolecular crowding favors compacted protein configurations, as they occupy less volume and are therefore thermodynamically preferred in crowded environments [14]. Since matter is impenetrable, macromolecules surrounding a protein decrease the real volume that the protein can occupy. This leads to an entropic destabilization of extended protein states (such as unfolded or unbound states). *In vitro* experiments support excluded volume theory: steric interactions stabilize compact states of proteins [15], slow protein diffusion, increase or decrease enzymatic activity [16–19], and increase protein assembly dynamics [20].

The second type of interaction, non-steric interactions, accounts for all other interactions between a protein and its environment. This includes interactions with small metabolites and ions, such as nucleotides, carbohydrates, cations, and anions, many of which are found at millimolar concentrations inside cells [22,23]. Non-steric chemical interactions arise from attractive and repulsive ionic interactions, hydrophobic interactions, and other non-covalent interactions with surrounding macromolecules and metabolites. This includes specific and nonspecific interactions with chaperones, ions, structural assemblies, nucleic acids, and lipid membranes. Such interactions are highly dependent on the surface characteristics and solvent-accessible surface area of the protein state. Therefore, non-steric interactions affect both compact and extended states, making it difficult to predict the net effect [15,24]. These interactions were mostly ignored until a decade ago when in-cell experiments demonstrated that surface interactions can suppress steric crowding effects [1,25–29]. This led to renewed consideration of how the cellular environment affects proteins.

A challenge of understanding steric and non-steric interactions in a cell is that, because both interactions are happening simultaneously, in-cell experiments only measure the net effect [5,6,30]. Furthermore, since the cellular environment is heterogeneous and in constant flux, steric and non-steric interactions modulate protein behaviors at the subcellular and organelle level [31–33]. For example, protein stability varies in the cytoplasm, nucleus, and ER [34] as well as upon cytoplasmic rearrangement [35,36]. Such results can be attributed to different steric and non-steric effects arising from local variations in pH, ion concentrations, and macromolecular compositions [37].

In recent years, to disentangle the combined effects of steric and non-steric interactions, *in vitro* experiments, using inert polymers, proteins, and buffers in systematic and well-controlled environments, have successfully interpreted *in cellulo* observations [5,6,15,24]. Here, we review recent efforts to accurately mimic the impact of the cellular environment on protein folding. We consider how mimetic recipes can be altered to account for differences between eukaryotes and prokaryotes, differentiate eukaryotic cell lines, and even distinguish subcellular compartments. Understanding how steric and non-steric interactions affect protein folding at a subcellular level is of particular importance, because it is now recognized that nearly all cellular processes are spatially organized and regulated within membrane-bound or membraneless compartments. Mimetics that accurately recapitulate such cellular effects are a cost-effective and simple method to predict in-cell protein behavior, such as to test drugs and therapeutic molecules that target specific areas of the cell [38–41].

## Steric crowding interactions

Early models of the cellular environment focused on steric crowding interactions, arising from the high concentrations of cellular macromolecules, as the primary modulator of in-cell protein folding. *In vitro* experiments that tested Minton’s proposed steric crowding model demonstrated that under crowded conditions, proteins are less likely to aggregate [42], more compacted in the unfolded state [43], fold to compact states more readily [44], and refold faster [45]. Additionally, Minton’s model predicts that crowding is most efficient when the crowder is similar in or smaller in size to the protein [46,47]. This is because small proteins can fit within the interfacial cavities between larger crowders, reducing or even eliminating entropic crowding effects. Consistent with this theory, the folding of small proteins and peptides is minimally affected by much larger crowding mimetics [5,6,48–50]. There are many reviews that discuss the physics behind macromolecular crowding and how it affects the thermodynamics and kinetics of protein folding [30,47,51,52]. Here, we focus on how steric crowding mimetics are being applied to study protein folding in a biologically relevant context, from organisms and tissues to organelles and subcellular compartments.

## Mimetics of steric crowding in organisms and tissues

Mass spectrometry studies estimate that *Escherichia coli* have between 300 and 400 mg/ml macromolecules, including proteins, lipids, and nucleic acids [53–55]. In eukaryotic cells the average concentration of macromolecules is much lower, 150–250 mg/ml [56–58], and proteins are on average ≈100 amino acids larger than in prokaryotes [59,60]. However, protein expression levels can vary widely by tissue type, even within the same organism. For example, eye lens cells are crowded with structural crystallin proteins in excess of 500 mg/ml [53]. Consistent with the excluded volume effect, the protein phosphoglycerate kinase (PGK) is stabilized and slowed by viscosity in the eye lens compared with other less crowded tissue types in live zebrafish [61]. Thus, *in vitro* mimetics of steric crowding interactions must be tuned to the specific cellular environment.

**Figure 2:**
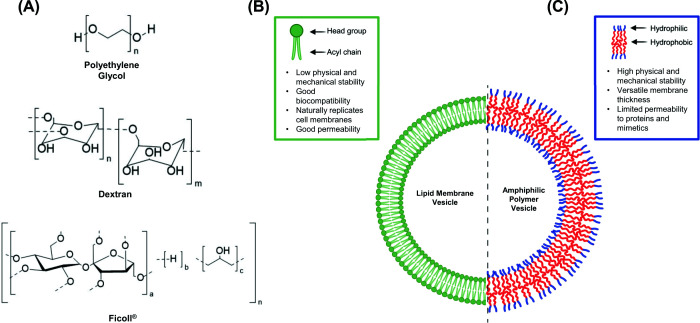
(A) Chemical structures of PEG, dextran, and Ficoll®. Schematic cartoon of a (B) phospholipid membrane and (C) polymer membrane GUV, comparing the different strengths and limitations for protein folding studies. Panel (A) created using ChemDraw. Panels (B) and (C) are created in BioRender.com and assembled in PowerPoint.

A good mimetic of steric crowding must (1) have limited non-steric chemical interactions, (2) be water soluble to ≈500 mg/ml, and (3) be available in high molecular weights to match cellular biomolecules. Relatively inert, highly soluble polymers (Figure 2A) such as dextran, Ficoll®, and polyethylene glycol (PEG) are commonly used as *in vitro* mimetics of cellular crowding [62]. Dextran and Ficoll® are polysaccharides; dextran is a linear glucose polymer, while Ficoll® is a branched copolymer of sucrose and epichlorohydrin. PEG is a polyether homopolymer comprised of ethylene glycol. While none are completely inert, Ficoll®’s branched polymer generates a more spherical structure that better matches the excluded volume of globular proteins than linear polymers [63,64].

Most often the effect of steric crowding is tested across a range of variables, including polymer identity, molecular weight, and concentration [5,6,15,24]. While the absolute properties of a protein vary between polymer identities, the excluded volume effect upon increased concentration typically holds. A simple mimetic of protein folding in eukaryotic cells might employ ≈150 mg/ml Ficoll70® [5,6,15], while a mimetic for prokaryotic cells would use ≈400 mg/ml PEG10K [65] to account for the difference in protein size and concentration. However, as no polymer is truly inert, there is no field consensus on the optimal recipe for an inert, steric crowding mimetic. Additionally, such monodispersed solutions will not achieve the same packing fraction as the polydispersed macromolecules found in native cellular environments [66]. In fact, a caveat of using these polymers is that they mesh at increasing concentrations, while the cell is crowded with compact globular proteins that jam at high concentrations [67]. Nevertheless, high concentrations of polymers may prove useful for mimicking confinement in mesh-like environments such as cytoskeletal lattices or extracellular matrix fibers [68], A few experimental studies have explored binary mixtures of crowding agents, which show nonadditive effects where the mixture is more stabilizing than the sum of the effects of the individual crowding agents [69–71]. Thus, polydispersity of crowders is an important variable that is often neglected when preparing simple monodispersed steric mimetics.

## Macromolecular crowding in sub-cellular compartments and transient organelles

Proteins are not homogenously polydispersed across the cell but rather organized into sub-cellular compartments and transient organelles with their own local concentration and polydistributions. Like eukaryotic protein concentrations, intranuclear macromolecular concentrations vary greatly, ranging from 170 to 400 mg/ml [72]. Many proteins are synthesized with localization tags that direct them to the compartments where they function. Furthermore, the distribution of steric interactions within a compartment can modulate the strength of the steric effect. For example, Davis *et al*. demonstrated that both the assembly and disassembly as well as local concentrations of the cytoskeleton can affect protein structure and stability [35]. For *in vitro* measurements with simple crowding mimetics, a FRET-based in-cell crowding sensor can be directed to the region of interest to estimate the local crowding effect [73].

Recently, more sophisticated steric crowding models that use artificial cells or organelles, consisting of biological material encapsulated in synthetic vesicles that mimic the cell membrane or membrane-bound organelles, have attracted interest [74]. Giant unilamellar vesicles (GUVs; Figure 2B,C) are the most common type of vesicle used to mimic biological membranes [75]. Inert polymers (e.g., PEG, Ficoll®, dextran) can be loaded into the vesicles with the protein of interest to mimic the combined effects of crowding and confinement on protein folding. The molecules used to create the membrane vary from phospholipids to amphiphilic polymers. Phospholipids are preferred due to their high biocompatibility as well as their ability to mimic natural cell membrane thickness (3–5 nm) and composition; however, they suffer from lower physical stability and are more sensitive to environmental stress (e.g., pH, temperature, and mechanical) compared with amphiphilic polymers [76]. For example, in a thermal denaturation experiment the phospholipid vesicle may break apart before reaching the protein’s melting temperature [76,77]. Amphiphilic polymers, on the other hand, have high thermal stabilities and can be used to study thermophilic proteins and protein complexes [78]. However, GUVs made from these polymers are limited in their permeability, making it difficult to introduce proteins and crowding mimetics into the vesicle. By tuning the mechanical properties of a synthetic vesicle with diblock copolymers, Jacobs *et al.* demonstrated that membrane protein folding is dependent on both steric interactions at the membrane surface and available membrane surface area [79]. In another study, Guerzoni *et al.* used lipid-based GUVs and Ficoll70® crowders to examine the effect of crowding on liposomes [80].

Alongside membrane-bound compartments, interest is growing in mimicking membraneless compartments *in vitro* [81]. These regions play important roles in both eukaryotic and prokaryotic cells and are characterized by their ability to create a high local concentration of, amongst other biomolecules, proteins [81]. Stable membraneless compartments include nucleoli, nuclear speckles, and Cajal bodies. Transient membraneless compartments, such as stress granules and P-bodies, assemble and disassemble dynamically to meet temporary local needs. Whereas classic membrane-bound organelles like endosomes, lysosomes, and transport vesicles are readily mimicked using vesicles made from lipid layers [82–84], membraneless compartments are not accurately mimicked using bound membranes. Therefore, much effort has been directed toward creating biocondensates *in vitro* and inside membrane-bound structures using the principles of liquid–liquid phase separation [85–88]. Mu *et al.* integrated membraneless coacervates, microdroplets of concentrated macromolecules like proteins or nucleic acids that are formed via liquid-liquid phase separation, into a proteinosome and demonstrated that enzymatic reactions are enhanced due to local enrichment of reactants in the droplets [89].

While GUVs and biocondensates are emerging as tools to mimic the crowded local environment of membrane-bound and membraneless organelles, it is important to note that lipids and concentrated condensates themselves are not inert. Non-steric protein–protein and protein–lipid interactions can affect not only the structure and function of the protein but also the structure of the lipid vesicle or condensate itself, which can make the interpretations of steric interaction effects ambiguous [90,91].

## Non-steric chemical interactions

Beyond steric crowding effects, the in-cell behavior of a protein is shaped by non-steric interactions. These encompass nonspecific and specific chemical interactions that a protein participates in with its surroundings that can be stabilizing or destabilizing; the resulting impact on a protein is dictated by the chemical composition of the local environment and the surface properties of the protein (Figure 1B) [30]. Although in-cell mimetics have historically prioritized crowding, it is becoming increasingly apparent that non-steric interactions are equally, and occasionally more, important modulators of in-cell protein folding. In fact, crowders themselves are not inert, and non-steric interactions modify their effective sizes. Thus, in moving towards biologically accurate *in vitro* mimetics, it is essential to account for non-steric chemical interactions. For example, the in-cell stability of small proteins and peptides that are significantly smaller than surrounding macromolecules is primarily determined by non-steric interactions [5,6]. The nature of such non-steric mimetics will necessarily vary with the cellular context being mimicked, given the diversity of proteome characteristics and metabolite compositions amongst different organisms and cell types and even among different cellular compartments within the same cell [92,93].

## Chemical composition of prokaryotes versus eukaryotes

The observation that surface charge differently modifies the diffusion properties of the same protein in bacterial and mammalian cellular environments suggests inherent differences in the types and distributions of chemical interactions available in prokaryotic and eukaryotic cells [94]. As such, the design of *in vitro* non-steric interaction mimetics must be tailored to the cellular setting.

Perhaps the simplest mimetic of non-steric chemical interactions is a buffer containing salt to replicate intracellular ionic strength. Salt modifies the chemical environment through electrostatic screening and the Hofmeister effect, which describes the mechanisms by which ions affect protein solubility [95–97]. Cytosolic ionic strength for mammalian cells is typically in the range of 150 mM and dominated by potassium ions [22,23]. Thus, 150–200 mM potassium chloride is often used to match the intracellular ionic strength of mammalian cells, but this value can be adjusted to reflect salt concentrations in the organism of interest [6,24,98]. This mimic was able to reproduce the in-cell expansion of variable major protein-like sequence expressed (VlsE) but has achieved limited success in accounting for in-cell stability trends [6,24].

Adding a layer of complexity, there also exist solutions that mimic the presence of small molecules and other cellular metabolites. Non-denaturing protein extraction reagents, such as Pierce™ IP Lysis Buffer and mammalian protein extraction reagent (M-PER™), are engineered to preserve protein structure and interactions using a combination of ionic strength, small organic molecules, and short-chain fatty acid mimics, although exact recipes are often proprietary. To provide an example of reagent composition, Pierce™ IP Lysis Buffer contains 25 mM Tris–HCl (pH 7.4), 150 mM NaCl, 1% NP-40, 1 mM EDTA, and 5% glycerol [99]. These reagents can be utilized as mimetics of nonspecific non-steric interactions in the cell [5,6,15,24]. Such reagents can effectively recapitulate cellular trends for proteins where crowding agents alone may not [15,24]; in particular, the stability of proteins that are significantly smaller than the surrounding biomolecules is dictated by non-steric chemical interactions [5]. Analogous protein extraction reagents for different prokaryotic and eukaryotic cell types can be house-made or are commercially available (e.g., B-PER™, BugBuster®, Pierce™, or M-PER™). Although the recipes of the reagents vary in concentration and chemical identity, they reliably predict nonspecific in-cell stability trends [65].

**Figure 3:**
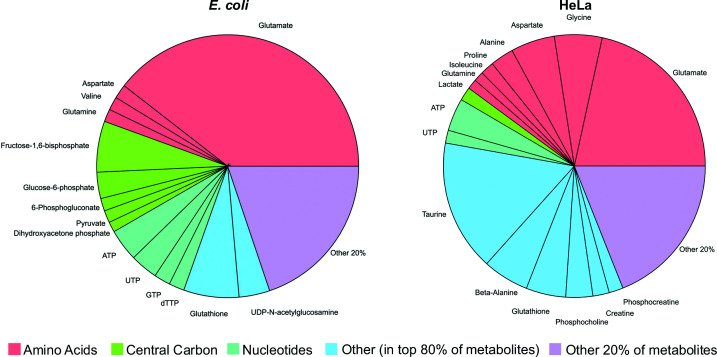
Metabolite composition of E. coli and HeLa cells. Metabolite composition of *E. coli* [93,101,102] and HeLa cells [100]. The top 80% of metabolites in each cell type, calculated in units of molarity, were used to construct Eco80 and HeLa80. Pi charts generated in IgorPro and assembled in PowerPoint.

However, prokaryotic and eukaryotic cells have specific metabolite compositions. Using metabolic data, an *in vitro* mimetic containing the top 80% of metabolites in *E. coli*, termed Eco80 (Figure 3), was constructed for RNA folding studies [93, 101, 102]. This was further applied to understand the folding of barnase, a bacterial ribonuclease, and inspired an equivalent mimetic based on metabolic data for HeLa cells [6,100]. The opposite stability trends observed for barnase in Eco80 and HeLa80, consistent with in-cell measurements, suggest the importance of quantitatively accounting for metabolite content in the relevant cellular environment.

Mimetics may also approach biological accuracy by incorporating the properties of larger cellular biomolecules. One example of this is using polyanions to reflect the abundance of anionic biomolecules in the cell, which better captured the in-cell destabilization of superoxide dismutase than small anions [103]. Low concentrations of proteins or DNA, below concentrations necessary to induce crowding effects, can also be used to predict nonspecific, non-steric interactions with macromolecules [6,25,104]. To capture specific, non-steric interactions, dilute lysates containing proteins and potentially other cellular contents extracted from a particular cell type have been widely used as an *in vitro* mimetic. Chymotrypsin inhibitor 2 is destabilized by chemical interactions in cytosolic extract from *E. coli*, with the extent of destabilization dependent on the concentration of protein in the extract [105]. Similarly, lysate from U2-OS or HeLa cells has been used to understand the in-cell folding and binding of diverse proteins [6,24,98,106,107]. Cell lysates are necessary to reproduce protein behaviors driven by specific protein–protein interactions inside cells [6,98]. Thus, while cell lysates are time-consuming and difficult to reproducibly prepare, they are the most reliable predictor of non-steric interactions.

## Non-steric interactions differ between eukaryotic cell lines and within cellular compartments

Though not as distinct as the environments of prokaryotic and eukaryotic cells, different eukaryotic cells may also differ chemically. For example, the proteomes of several common eukaryotic cell lines, including HeLa and U2-OS cells, have been experimentally determined [92,108,109]. These data show that while much of the proteome is conserved between cell lines, protein expression levels vary [92]. Thus, lysates should be prepared from the cell line of interest. Furthermore, this data offers an opportunity to construct cell-line-specific *in vitro* mimetics that replicate the protein composition of the respective cell line, serving as a protein-based equivalent to Eco80/HeLa80. The development of such mimetics would be further aided by the collection and inclusion of metabolic data for various types of eukaryotic cells.

Compartmentalization further divides eukaryotic cells into different chemical environments. Fluorescence imaging-based measurements demonstrate that protein stability and folding kinetics are dependent on subcellular localization [6,110]. Thus, mimetics of non-steric chemical interactions must account for local environmental differences. Given that intracellular ionic strength may deviate spatially, a FRET-based ionic strength sensor has been developed for in-cell measurement of ion concentrations [23]. Another avenue for replicating these environments *in vitro* is to use compartment-specific protein extraction reagents. For example, there are extraction reagents formulated for cytoplasmic and nuclear proteins; these can be used to mimic nonspecific chemical interactions in the respective compartment or to otherwise prepare cytoplasmic and nuclear lysates [6]. This approach has been applied to barnase, which was observed to have opposite stability trends in the cytoplasm and nucleus [6]. These trends were best reproduced using cytoplasmic and nuclear lysates, suggesting that its stability was determined by chemical interactions with the proteins in each compartment [6]. There also has been work on reconstituting the endoplasmic reticulum *in vitro* and probing its ion dynamics, which may allow for the development of an *in vitro* mimic for the folding of secretory proteins in this compartment [111–113]. Mimics for additional organelles require in-depth knowledge of their chemical environments.

## Conclusion and future directions

### Mimetics that account for steric and non-steric interactions

Various *in vitro* mimetics have been developed to untangle the individual importance of steric and non-steric interactions in modulating in-cell protein behavior. Moving forward, mimetics that simultaneously account for crowding and chemical interactions offer the potential to simply and accurately approximate cellular conditions. One approach is to examine the protein of interest within a homogenous protein concentrated to physiological crowding levels. Many early crowding studies used bovine serum albumin as a crowding agent [45,114]. Temperature-dependent fluorescence measurements were enabled in a crowding matrix of the low-fluorescence thermophilic protein SubL [115]. Alternatively, the protein of interest can be concentrated to physiological crowding levels to induce a phenomenon referred to as self-crowding. This has been performed with the B1 domain of streptococcal protein G (GB1) and revealed residue-specific impacts of steric and non-steric interactions on stability [116].

**Figure 4:**
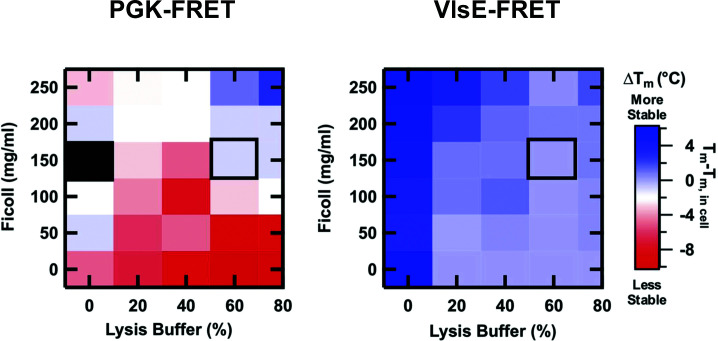
Two-dimensional scan of Ficoll and lysis buffer concentrations. Two-dimensional scan of Ficoll and lysis buffer concentrations, reporting the change in melting temperature (Δ*T*_m_) between varying *in vitro* conditions (*T*_m_ in the presence of different combinations of Ficoll and lysis buffer) and in-cell (*T*_m_, in cell) for PGK and VlsE. The black box outlines the *in vitro* condition that best reproduces the in-cell observation. Figure reproduced from [15].

Crowding and chemical interaction reagents can also be combined to generate more complex, cell-like mixtures. For example, Ficoll and Pierce™ lysis buffer exert nonadditive effects on protein stability, and a mixture of 150 mg/ml Ficoll and 60% lysis buffer exactly reproduced the opposite in-cell stability of PGK and VlsE (Figure 4) [15]. Later an improved, optically transparent recipe using 20% M-PER™ lysis buffer produced the in-cell stability of PGK, the fourU RNA thermometer [65], and a λ-repressor fragment [5]. While promising, the same mixture is less accurate and even fails when specific interactions play a large role in modulating the in-cell protein behavior [6,98].

Lysates are perhaps the most biologically relevant mimic, as they contain materials directly extracted from the cellular environment; however, concentrated lysates often become viscous and suffer from poor protein solubility. To address this, it was recently demonstrated that *E. coli* lysates can be concentrated to physiological levels using GUVs to match macromolecule concentrations of both prokaryotic (220–300 mg/ml) and eukaryotic cells (50–100 mg/ml) [117]. Such artificial cells provide a platform for understanding protein behavior in environments that capture both the steric and non-steric chemical interactions that exist in the native cellular environment.

### Opportunities for experimental and computational development

In the future, there is opportunity for the development of more specific in-cell mimetics, accounting for differences between various types of prokaryotic and eukaryotic cells, in addition to the numerous membrane-bound and membraneless compartments within the same cell type. Development of such mimetics remains limited by the low number of proteins that have been characterized inside cells. To validate new mimetics, additional quantitative in-cell data must be collected in the mimicked cell lines and subcellular compartments. Mimetic development additionally requires further metabolic and proteomic data to construct mimetics that accurately reproduce the composition of the local cellular environment. Similar work is occurring in the nucleic acid field [65,93,118] and crosstalk will accelerate the development of mimetics that benefit both fields [6,65].

Finally, a long-standing challenge in the field of molecular biology has been predicting protein structure from sequence alone. Recent advancements in experimental and computational approaches have resulted in deep learning models capable of high-accuracy sequence-based structure prediction [119–121]. However, prediction of protein behaviors inside cells is hampered by the limited availability of high-quality in-cell training sets. Cellular mimetics may aid in bridging the gap between theory and experiment in this field, providing a cost-effective and reproducible intermediate between *in vitro* and *in vivo*. It is essential that structural models consider the full proteomes of the protein of interest, as proteins do not act in isolation. Such context is key to understanding biological processes as well as disease and to the development of new drugs and therapies.

## Perspectives

- The cellular environment modulates protein folding and function. Simple *in vitro* mimetics that replicate the cell interior can be used to understand how proteins behave in their native settings and predict in-cell behaviors.
- It is widely accepted that the cell modifies protein properties via steric crowding and non-steric chemical interactions, and various mimetics have been developed to account for both.
- In the future, development of mimetics that incorporate metabolic and proteomic data for diverse cell types and compartments coupled with technological advancements in high-throughput methodologies will address issues with reproducibility and capacity of current *in vivo* protein folding experiments. Such approaches will produce datasets large enough to enable prediction of in-cell protein folding, which may be especially valuable for screening therapeutic candidates and for the advancement of sequence-based structure-prediction methods.

## Open Access

Open access for this article was enabled by the participation of Yale University in an all-inclusive *Read & Publish* agreement with Portland Press and the Biochemical Society.

## Abbreviations

**B-PER**™: bacterial protein extraction reagent
FRET: Förster resonance energy transfer
GB1: B1 domain of streptococcal protein G
GUV: giant unilamellar vesicle
IP: immunoprecipitation
**M-PER**™: mammalian protein extraction reagent
PEG: polyethylene glycol
PGK: phosphoglycerate kinase
VlsE: variable major protein-like sequence expressed
